# Perioperative management of thoracic surgery in patients with lymphangioleiomyomatosis

**DOI:** 10.1186/s40792-022-01507-5

**Published:** 2022-08-01

**Authors:** Mariko Fukui, Kuniaki Seyama, Takeshi Matsunaga, Aritoshi Hattori, Kazuya Takamochi, Shiaki Oh, Izumi Kawagoe, Kenji Suzuki

**Affiliations:** 1 Department of General Thoracic Surgery, Juntendo University School of Medicine, Tokyo, Japan; 2 Department of Respiratory Medicine, Juntendo University School of Medicine, Tokyo, Japan; 3 Department of Anesthesiology, Juntendo University School of Medicine, Tokyo, Japan

**Keywords:** Lymphangioleiomyomatosis, Thoracic surgery, Surgical outcome, Chylothorax, Pneumothorax

## Abstract

**Background:**

General surgery for patients with lymphangioleiomyomatosis (LAM) is infrequent, however, general surgeons also occasionally experience it. Only a few reports have described the specific perioperative management appropriate for patients with LAM. Hence, in this case series, we aimed to investigate the surgical outcomes of LAM patients and their characteristics.

**Case presentation:**

Medical records of 4482 patients who underwent thoracic surgery between 2009 and 2017 at our institution were assessed. Twelve patients were diagnosed with LAM. Details of the postoperative courses and surgical outcomes of LAM patients were retrospectively examined.

All LAM patients were female (age 41.3 ± 10.6 years). Surgeries were performed for patients undergoing biopsy (*n* = 4) and those with pneumothorax (*n* = 3), lung cancer (*n* = 2), and other conditions (*n* = 3). The mortality rate was 0% and the length of hospital stay was 27.4 ± 8.9 days. Ten postoperative complications occurred in six patients (50%): hypoxemia (*n* = 5), chylothorax (*n* = 2), and prolonged air leakage (*n* = 3).

**Conclusions:**

Thoracic surgery may pose a risk of postoperative complications and long hospitalization for patients with LAM, although it lowers the risk of fatality. Management of perioperative air and chyle leakages and lymphatic stasis in the lungs is important for preventing morbidities.

## Background

Lymphangioleiomyomatosis (LAM) is a systemic neoplastic condition in which the proliferation of smooth muscle-like neoplastic cells (LAM cells) leads to progressive cystic destruction of the lungs [1]. LAM cells infiltrate the pulmonary interstitium and obstruct the airways, lymphatics, and blood vessels. LAM occurs mainly in women aged 30–50 years, affecting 1.9–4.5 per million women in Japan [2] and 3.4–7.8 per million worldwide [3]. The disease slowly progresses with the development of symptoms, such as dyspnea, cough, and bloody sputum [2, 4]. Pneumothorax occurs in 43–70% of patients with LAM [2–5] and often recurs; therefore, it is necessary to pursue preventive strategies for recurrence, such as pleurodesis, surgery, and drainage, even for the first pneumothorax [6, 7]. Further, some institutes perform total pleural covering to prevent recurrence [8]. LAM patients are usually treated in specialized facilities, such as lung transplant facilities; however, general respiratory surgeons can also treat pneumothorax in LAM patients.

The administration of sirolimus has been reported to improve the respiratory status of patients with LAM [8]. Due to the slow progression of LAM and median transplant-free survival of over 20 years [5, 9], LAM patients tend to develop complications such as lung cancer and other lung infections, thereby increasing the need for surgery.

To date, only a few reports have described the specific perioperative management appropriate for patients with LAM. Hence, in this retrospective study, we aimed to investigate the surgical outcomes of LAM patients and their characteristics.

## Case presentation

The present study included patients with LAM who underwent thoracic surgery between January 2009 and December 2017. Out of 4,482 patients who underwent thoracic surgery, 13 patients were suspected to have or diagnosed with LAM. Out of these 13 patients, one patient had a postoperative pathological diagnosis inconsistent with LAM and was excluded. Finally, 12 patients, whose diagnoses were pathologically confirmed, were included in this study. Some clinical data of one LAM patient (Case 1 in Table 2) has already been reported in previous studies [10, 11]. However, we included this case since we focused on another aspect of clinical manifestation.

### Clinical characteristics of study population

There were 12 patients with LAM and all were females, with a mean age of 41.3 ± 10.6 years (Table 1). There were no cases with renal dysfunction or cardiac dysfunction. Table 2 shows a list of all 12 LAM patients with their perioperative clinical data. Among all the LAM patients, two patients who underwent surgery for lung cancer were 58 and 63 years old, while the age of other patients ranged from 27 to 49 years. All patients with lung cancer were adenocarcinoma, and intraoperative rapid pathology of the hilar lymph nodes confirmed the absence of metastases, thus omitting mediastinal lymph node dissection. The pathological stage was Stage IA in Case 10 and IIA in Case 11. All patients except three (Case 2, 11, and 12 in Table 2) never smoked. The mean partial pressure of arterial oxygen before surgery was 82.3 ± 12.3 mmHg. Preoperative pulmonary function tests were not performed in three patients who underwent surgery for pneumothorax and one patient who underwent surgery with drainage treatment for empyema. The forced expiratory volume in one second was > 70% of the predicted value in all cases. Two patients had angiomyolipoma (Cases 8 and 10 in Table 2), and three had a history of pneumothorax (Cases, 2, 3, and 11 in Table 2). Sealants were used in seven patients to cover the resection lines to prevent air leakage. However, three patients had prolonged air leakage despite the use of sealants.

**Table 1:** Clinical characteristics of study population

Variables	LAM
Number of patients	12
Sex (male/female)	0/12
Age (years) (mean ± SD)	41.3 ± 10.6
Surgical indication	
Biopsy for LAM	4 (33.3%)
Pneumothorax	3 (25.0%)
Lung cancer	2 (16.6%)
Empyema	1 (8.3%)
Others^a^	2 (16.6%)
Preoperative PaO_2_ (mmHg)	82.3 ± 12.3
Respiratory function ( *n* = 9)	
VC (L)	3.03 ± 0.40
%VC (%)	97.9 ± 12.9
FEV_1_ (L)	2.18 ± 0.36
FEV_1_/FVC (%)	76.01 ± 9.28
%DL_CO_ (%)	60.8 ± 16.1
Operative time (min)	73.8 ± 39.6
Operative blood loss (ml)	39.8 ± 75.6

*FEV*_*1*_ forced expiratory volume in 1 s, *FVC* forced vital capacity, *LAM* lymphangioleiomyomatosis, *PaCO*_*2*_ partial pressure of arterial carbon dioxide, *PaO*_*2*_ partial pressure of arterial oxygen, *%DL*_*CO*_ % of the predicted value of diffusing capacity of the lung for carbon monoxide, *%VC* % of the predicted value of VC, *SD* standard deviation, *VC* vital capacity

^a^Other surgical indications were aspergilloma and benign lung tumor (adenofibroma)

**Table 2:** Perioperative course of patients with lymphangioleiomyomatosis after thoracic surgery

No.	Age	AML	FEV_1_ (L)	PaO_2_ (mmHg)	PF ratio	Surgical indication	Procedure	Sealant	X-ray findings (contralateral)		Complications	Hospitalization (days)
				(FiO_2_)	Before surgery/5POD				CPA dull	Permeability decay		
1	36	−	1.94	70.3 (0.21)	334.8/296.7	Aspergilloma	Lobectomy	Oxidized cellulose, fibrin adhesive	+	+	Hypoxemia, Air^a^, Chylo^b^	95
2	38	−		98.5 (0.21)	469.0/–	Pneumothorax	WWR	fibrin adhesive	−	−		23
3	39	−		86.6 (0.21)	412.4/375.0	Pneumothorax	Suturing	PGA sheet, fibrin adhesive	−	+		15
4	27	−	2.64	97.3 (0.32)	304.1/500.6	Pneumothorax	WWR	PGA sheet, fibrin adhesive	−	+	Air^a^, Chylo^b^	25
5	38	−	2.84	65.7 (0.21)	312.9/144.7	Biopsy	WWR	PGA sheet	+	+	Hypoxemia	34
6	41	−	1.97	88.5 (0.21)	421.4/446.6	Biopsy	WWR	−	+	+		6
7	49	−	2.07	69.1 (0.21)	329.0/326.7	Biopsy	WWR	−	+	+		8
8	33	+	2.24	85.6 (0.21)	407.6/–	Biopsy	WWR	−	−	+		3
9	31	−		79.9 (0.32)	249.7/244.1	Empyema	Fenestration	−	+	+	Hypoxemia	71
10	58	+	1.86	98.2 (0.21)	467.6/353.3	Lung cancer	Segmentectomy	PGA sheet, fibrin adhesive	+	+		6
11	63	−	1.90	76.5 (0.21)	364.3/285	Lung cancer	Bilobectomy	−	+	+	Hypoxemia, Air^a^	18
12	42	−		70.0 (0.21)	333.3/248.6	Lung tumor	WWR	PGA sheet, fibrin adhesive	+	+	Hypoxemia	22

*Air*^a^, prolonged air leakage, *AML* angiomyolipoma, *Chylo*^b^ chylothorax, *CPA* costphrenic angle, *FiO*_*2*_ fraction of inspiratory oxygen, *PF ratio* the ratio of arterial oxygen partial pressure (PaO_2_ in mmHg) to fractional inspired oxygen (FiO_2_) expressed as a fraction, *PGA sheet* polyglycolic acid sheet, *WWR*, wide wedge resection

### Perioperative management

Patients were hospitalized the day before surgery and were required to fast after dinner. After rapid induction, total intravenous and epidural anesthesia with analgesia were administered. A double-lumen tracheal tube was used for single-lung ventilation, and the patients were extubated in the operating room immediately following surgery.

For lung tumors, we performed video-assisted thoracotomy via a 7- to 15-cm posterolateral skin incision without cutting through the ribs. For lung biopsy and pneumothorax surgery, we performed thoracoscopic pulmonary resection without pleurodesis via three ports. The in–out balance of infusion, urine, and blood was adjusted at a rate of 5–6 mL/kg/h during surgery. The postoperative fluid level was planned to be 1.3–1.5 mL/kg/h until the start of the meal at lunch the day after surgery. The thoracic drain was removed when the drainage volume was lower than 300 mL per day and no air leakage was observed. All patients were oxygenated and monitored using electrocardiography for at least 2 days postoperatively. Chest radiography was performed on the day of surgery and postoperative days (PODs) 1 to 5. Arterial blood gas analysis was performed after anesthesia and on POD 1. Laboratory findings were evaluated on PODs 1, 3, and 5.

### Surgical outcomes and postoperative course

Table 3 shows the surgical outcomes of the LAM patients. The mortality rate was 0% and the mean length of hospital stay of LAM patients was 27.4 ± 8.9 days. The longest hospital stay was 95 days (Case 1 in Table 2). At present, 9 of the 12 patients are taking sirolimus, but only the patient labelled as Case 1 has been taking sirolimus before surgery. In Case 1, sirolimus was discontinued since 2 weeks prior to 2 weeks after surgery. A total of 10 postoperative complications occurred in six patients (50%), eight of which were of Clavien–Dindo grade ≥ II. The most common complication was postoperative hypoxemia, which occurred in five patients who required oxygen administration after POD 2. Weight gain was observed in eight patients, of whom seven gained weights until PODs 3–5, while one continued to gain weight until POD 10.

**Table 3:** Surgical outcomes after thoracic surgery

Outcomes	LAM
Number of patients	12
Ninety-day mortality	0
Length of hospital stay (mean ± SD)	27.4 ± 8.9
Surgical complications (Clavien-Dindo grade ≥ II)	8
Arrythmias	0
Chylothorax	1 (8.3%)
Hypoxemia	5 (41.6%)
Air leakage	2 (16.6%)
*X-ray findings*	
Decreased radiolucency (POD 2)	11 (91.6%)
Decreased radiolucency (POD 5)	5 (41.7%)
Pleural effusion on the opposite side of the surgery (POD 2)	9 (75.0%)
Pleural effusion on the opposite side of the surgery (POD 5)	5 (41.7%)
Maximum weight gain after surgery	1.0 ± 0.9

*LAM* lymphangioleiomyomatosis, *POD* post-operative day

**Fig. 1:**
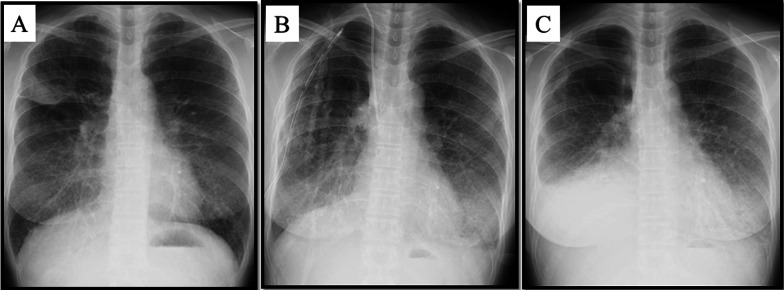
Chest radiograph findings of complications after thoracic surgery in a representative LAM patient (Case 1 in Table 2). **a** Preoperative chest radiograph shows aspergilloma in the right upper lung field. **b** Chest radiography findings 2 days after right upper lobectomy. A drain was inserted into the right thoracic cavity, and a central venous catheter inserted in the right internal jugular vein. Both are visible in the right lung field. This figure shows decreased radiolucency in bilateral lungs and pleural effusion on the opposite side of the surgery. **c** A chest radiograph 14 days after surgery shows that pulmonary congestion and pleural effusion are getting worse

The decreased radiolucency of the lung fields and contralateral pleural effusion on radiographs remained even on POD 5 in 41.6% of the LAM patients. (Table 3). Of the five cases with contralateral pleural effusions on POD5, only 1 case was diagnosed as a chylothorax by thoracentesis. Two cases were clinically diagnosed as chylothorax after diuretics failed to reduce the effusion and fat restriction reduced the effusion. One of two chylothorax cases were Grade II or higher chylothorax requiring medical treatment (Table 3). Figure 1 shows changes in postoperative chest radiography findings of the patient represented as Case 1. On POD 2, the radiolucency of bilateral lungs had decreased and pleural effusion appeared in the side contralateral to the side that underwent surgery (Fig. 1b), the characteristics of which were particularly intense in the representative LAM case. On POD 14, radiograph of the LAM case showed blunting of bilateral costophrenic angles and decreased radiolucency in bilateral lower lung fields, which worsened (Fig. 1c).

Five patients were managed by the Department of Respiratory Medicine (Cases 1, 4, 8, 9, and 11 in Table 2) and underwent a fat-restricted diet in the perioperative period. Patient 11, who underwent bilobectomy, fasted from the day before surgery until 2 days after surgery. Fat restriction started from the start of meals after surgery and continued until about 1 month after discharge.

The length of hospital stay at our facility of six patients was ≤ 7 days and that of the remaining six patients was ≥ 10 days. The patients who required an extended hospital stay had prolonged chylothorax and hypoxemia and had to remain hospitalized for sufficient rest, fat restriction, and oxygen administration.

## Discussion

To the best of our knowledge, this is the first case series study focusing on the perioperative management of thoracic surgery in patients with LAM. Some reports on the surgical outcomes of lung transplantation for LAM are available [2, 12, 13]; however, literature on the operative management of LAM involving general thoracic surgery is limited. In this study, we aimed to investigate pertinent and appropriate perioperative management strategies for LAM, including thoracic surgery, which can equip general thoracic surgeons to manage this condition.

In this study, patients with LAM had many complications and long hospital stay. The frequency of surgical complications in LAM patients was found to be > 50%. A randomized controlled trial on segmentectomy and lobectomy in Japan reported the postoperative surgical outcomes for early-stage lung cancer and that the frequency of early complications was 22.7% [14]. The median age of the patients was 67 years, and all patients underwent segmentectomy or lobectomy. Patients with LAM in this study had many complications, despite their young age (median age: 38.5 years) and having undergone less surgical invasion, i.e., more than half of the patients underwent bullectomy or partial surgery. Thus, this high morbidity rate and pathophysiological features of lungs with LAM should be considered when determining surgical strategies for patients with LAM.

There are three types of postoperative complications of LAM: prolonged air leakage, chylothorax, and hypoxemia. Early surgical complications after lung transplantation in patients with LAM are reported to be graft dysfunction (17.2%), acute rejection (20.7%), pneumothorax (24.1%), and chylous effusion (20.7%) [12], of which graft dysfunction and acute rejection are transplant-specific. Postoperative occurrence of pneumothorax and chylous effusion appear to be common complications and characteristic of LAM, considering that they were also found in our study. These two postoperative complications are known to be closely associated with pathophysiologic features of LAM lungs, i.e., numerous fragile thin-walled cysts and abundant LAM-associated lymphatics in the visceral pleura and lung parenchyma. Therefore, a lung with LAM must be handled with great care and caution.

Prolonged air leakage has been reported in approximately 10–24% cases of post-transplant complications [12, 15, 16]. It is worth noting that pneumothorax can occur not only in the operated lung but also in the contralateral lung. In the management of pneumothorax, the use of nitrous oxide should be avoided and the airway pressure during intraoperative ventilation should be kept low. Pain control and depth of anesthesia should warrant extra care in pneumothorax cases. Protective operations are required, and sealants may be used to prevent air leakage from the resection line.

Chylothorax in patients without LAM has been reported to occur in 2.6% of patients who underwent lung cancer resection with lymph node dissection [14] and has a frequency of 10–30% in cases with post-transplant complications [17, 18]. In this study, approximately 16.6% of patients with LAM developed chylothorax. Chylothorax after transplantation or resection of lung cancer is caused by a lymphatic fistula due to lymphatic vessel damage. It is treated with a fat-restricted diet, adhesion therapy, and thoracic duct ligation. Contrarily, it is widely mentioned in the literature that chylothorax in LAM patients results from the blockage of the thoracic duct and its branches by LAM cells or the transdiaphragmatic flow from chylous ascites [19, 20]. The effectiveness of thoracic duct ligation as treatment is reported to be unclear and management of chylothorax in patients with LAM should be individualized depending on the size and clinical effects of the chylous pleural effusion, as well as comorbidities and the surgeon’s expertise [20]. In our study, procedures involving the thoracic duct were not performed. Furthermore, no LAM patients had chylous ascites. Case 1 in this study had diffuse lymphatic congestion in the lungs prior to initiating sirolimus therapy, which had disappeared since the initiation of sirolimus. Thus, we speculate that the appearance of chylothorax and hypoxemia could be re-activation of underlying LAM pathophysiology after discontinuation of sirolimus and possibly due to surgical manipulation of LAM lung. However, the precise mechanisms of postoperative chyle leakage into the pleural space remain elusive in this study. We speculate that abundant LAM-associated lymphatics on the visceral pleura and lung parenchyma might be a potential source of lymph leakage during surgery.

Prolonged postoperative hypoxemia is also a characteristic problem in patients with LAM. Postoperative exacerbation of lymphatic stasis may cause hypoxia after surgery. In general, the circulating plasma volume increases 1–3 days after lung resection because the plasma component that leaked into the stroma secondary to surgical intervention returns to the blood vessels. This fluctuation in circulating plasma volume is not significant in most cases because of the compensatory mechanisms of homeostasis, such as an increase in urine volume. Further, this can be easily managed with the control of fluid volume and diuresis. However, pulmonary congestion is more likely to persist postoperatively in patients with LAM due to stasis in the abundant lymphatic vessels in the LAM lungs. This prolonged pulmonary congestion may have influenced the prolonged decrease in lung permeability on postoperative x-rays and weight gain in the present study. Furthermore, LAM patients are prone to hypoxemia due to pulmonary congestion because of decreased diffusion capacity caused by cystic destruction of the lung parenchyma. Fat restriction and rest have been reported as management for this condition, with efficacy extending postoperatively [21]. Controlling LAM is also important to treat hypoxemia. It has been reported that administration of sirolimus improved lung permeability on computed tomography (CT) [10, 22]. The radiolucency on the X-ray in this study is the same phenomenon as permeability on CT, and may be considered as indicator of lymphatic flow stasis. However, wound healing has been reported to be impaired as a side effect of sirolimus. In Case 1 in our study, discontinuation of sirolimus contributed to the development of hypoxemia and chylous pleural effusion. At present, all lung transplantation centers in Japan consider it mandatory for LAM patients to quit taking sirolimus prior to lung transplantation [23]. This is because airway anastomosis dehiscence, a fatal complication of lung transplantation, has been reported to occur when sirolimus-based immunosuppressive therapy was initiated immediately after transplantation [24, 25]. Currently, the optimal perioperative withdrawal period of sirolimus remains undermined. Further studies are needed to establish this by assessing factors, such as the degree of surgical invasiveness, dose and length of sirolimus treatment, and severity of LAM.

## Conclusion

Thoracic surgery for patients with LAM is likely to have a higher risk of postoperative complications and long-term hospitalization. Perioperative management strategies, such as fat reduction prior to surgery and continual observance for perioperative pneumothorax, chyle leak, and lymphatic stasis, should be adopted to reduce complications.

## Data Availability

The datasets generated during and/or analyzed during the current study are not publicly available due to privacy policy in our institute but are available from the corresponding author on reasonable request.

## Abbreviations

FEV_1_: Forced expiratory volume in one second
FVC: Forced vital capacity
LAM: Lymphangioleiomyomatosis
PaCO_2_: Partial pressure of arterial carbon dioxide
PaO_2_: Partial pressure of arterial oxygen
POD: Post-operative day
%DL_CO_: % Of the predicted value of diffusing capacity of the lung for carbon monoxide
%VC: % Of the predicted value of VC
SD: Standard deviation
VC: Vital capacity
